# Impact of Gram stain results on initial treatment selection in patients with ventilator-associated pneumonia: a retrospective analysis of two treatment algorithms

**DOI:** 10.1186/s13054-017-1747-5

**Published:** 2017-06-19

**Authors:** Jumpei Yoshimura, Takahiro Kinoshita, Kazuma Yamakawa, Asako Matsushima, Naoki Nakamoto, Toshimitsu Hamasaki, Satoshi Fujimi

**Affiliations:** 1 Division of Trauma and Surgical Critical Care, Osaka General Medical Center, 3-1-56 Bandai-Higashi, Sumiyoshi-ku, Osaka 558-8558 Japan; 2 Department of Advanced Acute Medicine, Nagoya City University Graduate School of Medical Sciences, 1 Kawasumi, Mizuho-cho, Mizuoho-ku, Nagoya-shi, Aichi 467-0001 Japan; 3 Office of Biostatistics and Data Management, National Cerebral and Cardiovascular Center, 5-7-1 Fujishirodai, Suita, Osaka 565-8565 Japan

**Keywords:** VAP, Gram staining, Antimicrobial therapy, Empirical therapy, Treatment bundle, Nosocomial infection, Mechanical ventilation, ICU, MDR, Favor method

## Abstract

**Background:**

Ventilator-associated pneumonia (VAP) is a common and serious problem in intensive care units (ICUs). Several studies have suggested that the Gram stain of endotracheal aspirates is a useful method for accurately diagnosing VAP. However, the usefulness of the Gram stain in predicting which microorganisms cause VAP has not been established. The purpose of this study was to evaluate whether a Gram stain of endotracheal aspirates could be used to determine appropriate initial antimicrobial therapy for VAP.

**Methods:**

Data on consecutive episodes of microbiologically confirmed VAP were collected from February 2013 to February 2016 in the ICU of a tertiary care hospital in Japan. We constructed two hypothetical empirical antimicrobial treatment algorithms for VAP: a guidelines-based algorithm (GLBA) based on the recommendations of the American Thoracic Society-Infectious Diseases Society of America (ATS-IDSA) guidelines and a Gram stain-based algorithm (GSBA) which limited the choice of initial antimicrobials according to the results of bedside Gram stains. The GLBA and the GSBA were retrospectively reviewed for each VAP episode. The initial coverage rates and the selection of broad-spectrum antimicrobial agents were compared between the two algorithms.

**Results:**

During the study period, 219 suspected VAP episodes were observed and 131 episodes were assessed for analysis. Appropriate antimicrobial coverage rates were not significantly different between the two algorithms (GLBA 95.4% versus GSBA 92.4%; *p* = 0.134). The number of episodes for which antimethicillin-resistant *Staphylococcus aureus* agents were selected as an initial treatment was larger in the GLBA than in the GSBA (71.0% versus 31.3%; *p* < 0.001), as were the number of episodes for which antipseudomonal agents were recommended as an initial treatment (70.2% versus 51.9%; *p* < 0.001).

**Conclusions:**

Antimicrobial treatment based on Gram stain results may restrict the administration of broad-spectrum antimicrobial agents without increasing the risk of treatment failure.

**Trial registration:**

UMIN-CTR, UMIN000026457. Registered 8 March 2017 (retrospectively registered).

## Background

Ventilator-associated pneumonia (VAP) is a serious healthcare-associated infection, common in intensive care units (ICUs). It complicates the medical course of 10–20% of mechanically ventilated patients and results in an estimated attributable mortality of 15–50% [1–3]. The selection of initial antimicrobial therapy for VAP is important since inappropriate initial antimicrobial treatment is associated with higher mortality and longer ICU stay [4–8]. Therefore, the 2005 American Thoracic Society-Infectious Diseases Society of America (ATS-IDSA) guidelines on healthcare-associated pneumonia recommend that empirical treatment of patients at risk of multi-resistant organisms should be broad spectrum to cover both multidrug-resistant (MDR) Gram-negative pathogens and methicillin-resistant *Staphylococcus aureus* (MRSA) [9].

Although early broad-spectrum treatment helps to ensure that infections are treated effectively, overuse of broad-spectrum antimicrobial agents is driving antimicrobial resistance [10, 11]. As VAP caused by multi-resistant Gram-negative bacilli results in significantly higher mortality than VAP caused by other pathogens [12, 13], preventing the emergence of resistant pathogens in the ICU is essential. One of the strategies which could potentially curtail the development of antimicrobial resistance is to use narrow-spectrum antimicrobial agents. In fact, national programmes aimed at combining judicious overall use of antimicrobial agents with narrow-spectrum agents have been associated with reductions in antimicrobial resistance [14, 15]. However, there are no well-established methods to safely restrict use of broad-spectrum antimicrobial agents for VAP.

While a culture of endotracheal aspirate is the gold standard for confirming the causative organisms of VAP, it takes at least 48–72 h to obtain the results of antimicrobial susceptibility tests. On the other hand, a Gram stain of respiratory specimens can provide immediate information about predicted pathogenic bacteria. Several studies have demonstrated the effectiveness of the Gram stain of endotracheal aspirate for diagnosing VAP [16–19]. However, its effectiveness in predicting causative organisms and guiding appropriate initial antimicrobial therapy has not been well established.

In our ICU, Gram stains of endotracheal aspirates are routinely performed by attending physicians to help diagnose VAP. However, the results of Gram stains have not been used to guide initial antimicrobial therapy. Thus, this study aimed to determine whether the Gram stain of endotracheal aspirate was a reliable guide for selecting appropriate antimicrobial therapy for VAP.

## Methods

### Study population

**Fig. 1:**
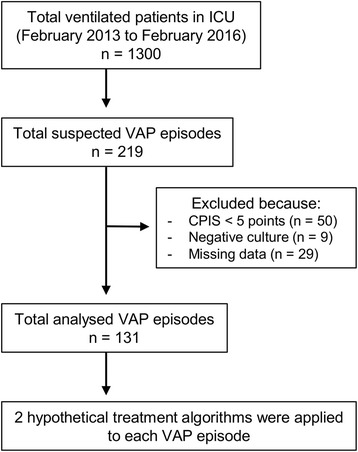
Patient diagram. *CPIS* clinical pulmonary infection score, *ICU* intensive care unit, *VAP* ventilator-associated pneumonia

This was a retrospective study conducted from February 2013 to February 2016 in the 18-bed ICU of a tertiary care hospital in Japan. Every patient admitted to our ICU during the study period was considered for the study if receiving mechanical ventilation for more than 2 days. Patients who were treated for VAP were eligible for this study. In order to select patients with a likely diagnosis of VAP, we included only those patients who had a modified clinical pulmonary infection score (CPIS) of 5 or more [20] and from whom semi-quantitative growth of a respiratory pathogen using a respiratory sample was estimated to be at least 1+. Patients with missing data relating to Gram stain or sputum culture were excluded. Only the first episode of suspected VAP was included (Fig. 1).

### Management

Methods for preventing VAP were based on the guidelines published by the Society for Healthcare and Epidemiology of America (SHEA) [21]: we managed patients without sedation whenever possible; we assessed readiness to extubate daily; we changed the ventilator circuit only if visibly soiled or malfunctioning; and we elevated the head of the bed to 30–45° if not contraindicated.

In our institute, Gram stain of endotracheal aspirate was performed on every patient with suspected VAP and the respiratory sample was sent to a laboratory for sputum culture. Gram stain was performed by the Favor method: heat-fixed smears on slides were flooded with 0.2% Victoria blue for 30 s and then washed with tap water; smears were decolourized with 2% Picric Acid ethanol; cells were counterstained with 0.004% Fuchsin for 30 s and then washed with tap water. Residents trained in this method performed a Gram stain as soon as possible after the respiratory sample was collected. Attending physicians evaluated physical findings, chest radiographs, and the results of Gram stains in reaching a diagnosis of VAP and determined an initial treatment strategy. Broncho-alveolar lavage (BAL) is not systematically performed at the study ICU.

### Development of the algorithms

To evaluate whether the Gram stain of endotracheal aspirate was a reliable guide for the selection of antimicrobial therapy for VAP we constructed two hypothetical empirical antimicrobial treatment algorithms: a guidelines-based algorithm (GLBA) and a Gram stain-based algorithm (GSBA).

**Fig. 2:**
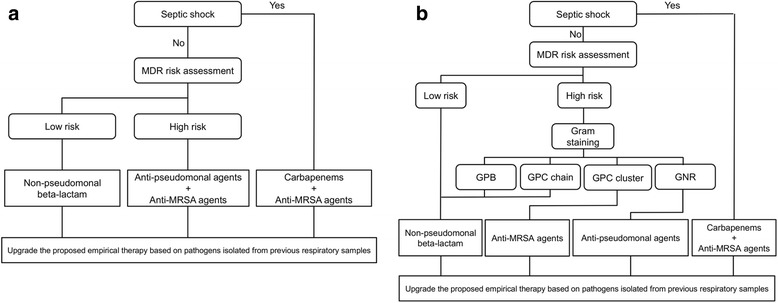
Algorithms of initial treatment selection. **a** *GLBA* guidelines-based algorithm, *GPB* Gram-positive bacilli, *GPC* Gram-positive cocci, *GNR* Gram-negative rods, **b** *GSBA* Gram stain-based algorithm, *MDR* multidrug-resistant, *MRSA* methicillin-resistant *Staphylococcus aureus*

In the GLBA (Fig. 2a), patients were initially assessed to determine whether they were in septic shock or not. A combination of a carbapenem and an anti-MRSA agent was selected to treat patients in septic shock. If patients did not present with septic shock, initial antibiotics were selected on the basis of the clinical risk factors for MDR pathogens according to ATS-IDSA guidelines [9]. The risk factors were defined as antimicrobial therapy in the preceding 90 days, a hospital stay of 96 h or more, chronic dialysis, immunosuppressive disease or therapy, nursing home admission, or colonizing MDR pathogens from surveillance cultures of endotracheal aspirate collected once a week. If patients had no MDR risk factors, a non-pseudomonal beta-lactam antibiotic was selected as an initial therapy. If patients had at least 1 of the MDR risks, the combination of an anti-pseudomonal agent and an anti-MRSA agent was selected. Finally, if drug-resistant pathogens were isolated from respiratory samples collected before the onset of VAP, we escalated an initial treatment selection process to cover them.

In the GSBA (Fig. 2b), the combination of a carbapenem and an anti-MRSA agent was selected for patients in septic shock, and the risk of MDR pathogens was assessed for patients not in septic shock using the same procedure as with the GLBA. If patients had no MDR risk factors, a non-pseudomonal beta-lactam antibiotic was selected as an initial therapy. If they had at least 1 of the MDR risks, results of bedside Gram stain of endotracheal aspirate were used to guide selection of initial treatment. The results of bedside Gram stains were categorized as Gram-positive cocci (GPC) chains, Gram-positive bacilli (GPB), GPC clusters, Gram-negative rods (GNR), or a combination of these. A non-pseudomonal beta-lactam antibiotic was selected for patients when the Gram stain of the endotracheal aspirate showed only GPC chains and/or GPB. An anti-MRSA agent was selected for patients when the Gram stain results showed GPC clusters without GNR. An anti-pseudomonal agent was selected for patients when the Gram stain results showed GNR without GPC clusters. The combination of an anti-pseudomonal agent and an anti-MRSA agent was selected for patients when the Gram stain results showed both GPC clusters and GNR. We escalated an initial treatment selection process to cover pathogens isolated from respiratory samples collected before the onset of VAP if the Gram stain results suggested their involvement.

In both algorithms, specific antimicrobial agents were selected according to previously recorded antimicrobial resistance patterns in our ICU.

### Outcome measures

Both the GLBA and the GSBA were hypothetically applied to the same VAP episodes and the antimicrobial agents proposed by each algorithm were compared retrospectively. Therapy was considered appropriate when all pathogens involved in the VAP episode were covered by antimicrobial agents. We defined the primary endpoint as the coverage rates of initial antimicrobial therapies. Secondary endpoints were set as the selected rates of anti-pseudomonal agents and anti-MRSA agents.

### Statistical analysis

Continuous variables were described as mean (± standard deviation) or median (interquartile range) for normal or non-normal distributions, respectively. To compare paired proportions, McNemar’s test for related samples was used. Differences in distribution were checked using the Wilcoxon signed-rank test.

All hypotheses for statistical tests were two-sided, and *p* values <0.05 were considered to indicate statistical significance. Statistical analyses were performed with R software (version 3.0.2; R Development Core Team) for Windows®.

## Results

### Patients

Figure 1 shows a patient diagram. There were 219 consecutive suspected VAP episodes during the study period, and 131 of them were used for analysis. The baseline characteristics of the study population are shown in Table 1. The median age of the patients was 67 years (range 48–80 years), and 87 (66.4%) were male. Clinical risk factors for MDR pathogens were present in 89 (67.9%) of the VAP episodes as follows: antimicrobial therapy in the preceding 90 days in 66 (50.4%), a hospital stay of 5 days or more in 70 (53.4%), chronic dialysis in 7 (5.3%), immunosuppressive disease or therapy in 6 (4.6%), nursing home admission in 7 (5.3%), and colonizing MDR pathogens from surveillance cultures of endotracheal aspirate in 17 (13.0%). The median Acute Physiology and Chronic Health Evaluation (APACHE) II score was 25 (range 19–30).

**Table 1:** The baseline characteristics of the study population

Number of patients	131
Age (years)	67 (48–80)
Male	87 (66.4%)
Diagnosis on hospital admission	
Trauma	56 (42.7%)
Sepsis	22 (16.8%)
Post-cardiac arrest syndrome	17 (13.0%)
Severe acute pancreatitis	8 (6.1%)
Burns	7 (5.3%)
Other	21 (16.0%)
APACHE II score	25 (19–30)
Risk factors for MDR pathogens	89 (67.9%)

Data are expressed as group median (interquartile range) or *n* (%)

*APACHE* Acute Physiology and Chronic Health Evaluation, *MDR* multidrug-resistant

### Appropriateness and spectrum of antimicrobial therapy

**Fig. 3:**
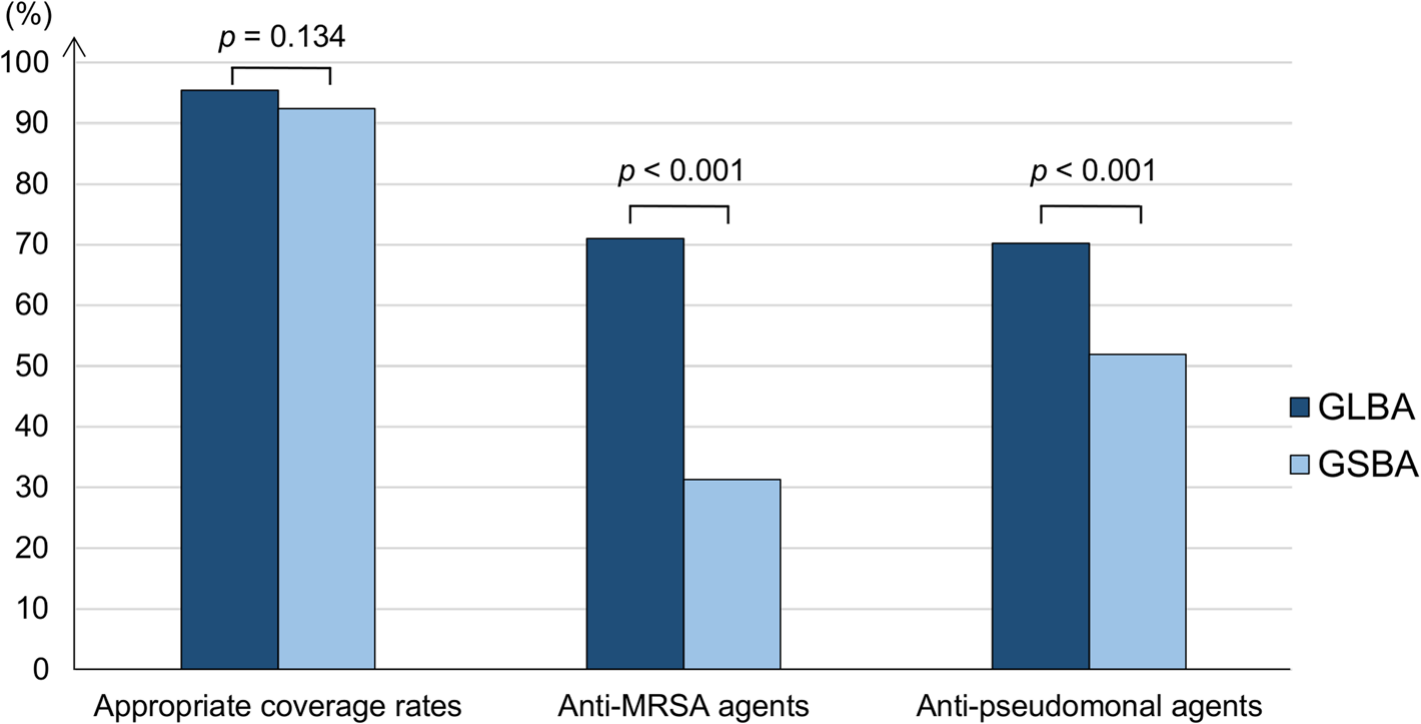
Appropriateness and spectrum of antimicrobial therapy. *GLBA* guidelines-based algorithm, *GSBA* Gram stain-based algorithm

Antimicrobial choices proposed by the GLBA and the GSBA were appropriate in 95.4% and 92.4% of cases, respectively. McNemar’s test showed no significant difference in adequacy for the different strategies (*p* = 0.134). The GSBA proposed anti-MRSA agents in significantly fewer episodes than the GLBA (41 (31.3%) versus 93 (71.0%), respectively; *p* < 0.001). The GSBA also recommended antipseudomonal agents in significantly fewer episodes than the GLBA (68 (51.9%) versus 92 (70.2%), respectively; *p* < 0.001) (Fig. 3).

Twenty-two MDR pathogens defined by the Centers for Disease Control and Prevention criteria were isolated: two extended spectrum beta-lactamase (ESBL)-positive enterobacteriaceae and 20 MRSA. In the GLBA, there were six inappropriate antimicrobial choices (4.6%): in five patients with no MDR risk factors, ampicillin sulbactam-resistant GNR was isolated (3.8%); in the other patient with at least one MDR risk factor, one ESBL-positive *E. coli* (0.8%) was isolated. Using the GSBA, 10 inappropriate antimicrobial choices were observed (7.6%): in five patients without any MDR risk factors, non-fermenter pathogens and MRSA were isolated (3 (2.3%) and 2 (1.5%), respectively); in the other five patients with at least one MDR risk factor, one ESBL-positive *E. coli* (0.8%) and one drug-resistant *Corynebacterium* sp. (0.8%) were isolated. Inappropriate antimicrobial choices due to misidentification of pathogens in the Gram-stained aspirate were observed in only three cases (2.3%), misidentification of GNR accounted for one case, and the other two were caused by misidentification of GPC clusters.

### Pathogens and Gram stain

From the 131 endotracheal aspirates that were Gram stained and cultured, 209 pathogens were isolated. Details of pathogens isolated are shown Table 2.

**Table 2:** Pathogens associated with ventilator-associated pneumonia

Pathogen	Number of patients (%)
Gram-positive bacteria	103 (78.6%)
*Staphylococcus aureus*	59 (45.0%)
MRSA	20 (15.3%)
*Streptococcus pneumonia*	4 (3.1%)
Other streptococci	44 (33.6%)
*Corynebacterium* sp.	15 (11.5%)
Gram-negative bacteria	75 (57.3%)
*Klebsiella* sp.	15 (11.5%)
*Haemophilus influenza*	14 (10.7%)
*Enterobacter* sp.	11 (8.4%)
*Escherichia coli*	10 (7.6%)
*Citrobacter* sp.	5 (3.8%)
ESBL-producing enterobacteriaceae	2 (1.5%)
AmpC-producing enterobacteriaceae	1 (0.8%)
*Pseudomonas aeruginosa*	24 (18.3%)
*Acinetobacter baumannii*	2 (1.5%)
*Serratia marcescens*	2 (1.5%)
*Stenotrophomonas maltophilia*	1 (0.8%)
Other Gram-negative bacteria	4 (3.1%)

*ESBL* extended spectrum beta-lactamase, *MRSA* methicillin-resistant *Staphylococcus aureus*

GPC clusters were identified on Gram stain in 64 patients, and *S. aureus* was cultured in 50 (78.1%) of these samples. Of the 67 patients from whom GPC clusters were not identified on Gram stain, *S. aureus* was cultured in nine samples (13.4%). The false-negative rates of GPC clusters were 15.3% (95% confidence interval (CI) 0.072–0.270) (Table 3). However, Gram-negative pathogens were cultured in 71 samples (74.7%) taken from 95 patients from whom GNR was detected on Gram stain. Gram-negative pathogens were cultured in four samples (6.7%) from 60 patients from whom GNR were not detected on Gram stain. The false-negative rates of GNR were 5.3% (95% CI 0.015–0.131) (Table 4).

**Table 3:** Results of Gram stain compared with *Staphylococcus aureus* culture

	*Staphylococcus aureus* growth in culture^a^		
GPC clusters on Gram stain	Yes	No	Total
Yes	50	14	64
No	9	58	67
Total	59	72	

^a^ Sensitivity of Gram stain: 50/59 = 84.7%; specificity of Gram stain: 58/72 = 80.6%; negative predictive value: 58/67 = 86.6%; positive predictive value: 50/64 = 78.1%

GPC Gram-positive cocci

**Table 4:** Results of Gram stain compared with GNR culture

	GNR growth in culture^a^		
GNR on Gram stain	Yes	No	Total
Yes	71	24	95
No	4	32	36
Total	75	56	

^a^ Sensitivity of Gram stain: 71/75 = 94.7%; specificity of Gram stain: 32/56 = 57.1%; negative predictive value: 32/36 = 88.9%; positive predictive value: 71/95 = 74.7%

*GNR* Gram-negative rods

## Discussion

The present study focused on the effectiveness of Gram stain to guide initial treatment selection in patients with VAP. The GSBA had a satisfactorily high coverage rate and suggested significantly lesser use of anti-MRSA agents and anti-pseudomonal agents than did the GLBA. Thus, the main findings were that the treatment algorithm constructed on the basis of Gram stain results could reduce the use of broad-spectrum antimicrobials without increasing the risk of treatment failure.

The guidelines for management of adult VAP published by the Infectious Diseases Society of America (IDSA) [22] have emphasized the importance of reducing the unnecessary use of broad-spectrum antimicrobials in order to minimize patient harm and reduce the development of antimicrobial resistance. Gram stain is a potentially useful tool to limit empirical broad-spectrum antimicrobial therapy in patients with VAP because it can provide information on predicted causative organisms promptly. However, whether Gram stain is accurate enough for the use of broad-spectrum antimicrobials to be safely restricted is still controversial. To the best of our knowledge, this study provides the first evidence that Gram stain of endotracheal aspirates may reduce the use of broad-spectrum antimicrobials without lowering appropriate coverage rates compared to a guidelines-based method.

In this study, the false negative rates of Gram stain were low both for GPC clusters (15.3%, 95% CI 0.072–0.270) and GNR (5.3%, 95% CI 0.015–0.131). The results of our study are consistent with previous studies that have reported high sensitivity and low false-negative rates for Gram stain used to identify *Staphylococcus aureus* [23–25]. On the other hand, some studies have reported only a fair correlation between Gram stain and culture [16, 26]. In these studies, some of the patients did not actually have VAP and might have had low-level colonization. Besides, the timing of the collected endotracheal aspirate was not clearly reported. In the present study, we only included confirmed VAP patients with modified CPIS of 5 or more and patients who had a high level of colonization—at least 1+ semiquantitative growth. In addition, all endotracheal aspirates were collected before the administration of the antimicrobials. These differences might have led to the considerably better correlation between Gram stain and culture in the current study. Moreover, a misidentification of GPC clusters from Gram stain did not necessarily result in insufficient coverage as selected antibiotics could cover methicillin-susceptible *S. aureus*. Similarly, misidentification of GNR was not detrimental unless non-fermenters or MDR pathogens were cultured. Therefore, we observed quite high appropriate coverage rates using the GSBA.

Several limitations have to be addressed. First, as this was a retrospective analysis of two hypothetical algorithms, the performance of both algorithms in reality could be different from the observed results. However, the findings from the Gram stains were independently confirmed by the final results from cultures of endotracheal aspirates. The superiority of the GSBA over the GLBA was derived from the high concordance rates between the results of Gram stain and sputum culture, indicating that, despite the retrospective study design, the Gram-stain diagnostic method would have high efficacy in clinical practice. Second, as the risk of inappropriate antimicrobial choices due to false-negative Gram stain results depends on the resistance rates of causative organisms, the coverage rates of GSBA alter according to local susceptibility in each ICU. Third, we could not evaluate whether patient outcomes from the GSBA were superior to the GLBA. Thus, a prospective study must be conducted to assess patient outcomes from antimicrobial treatment based on Gram stain results.

## Conclusions

Compared with guidelines-based treatment, bedside Gram stains may reduce the use of broad-spectrum antimicrobials for patients with VAP without lowering appropriate coverage rates.

## Abbreviations

APACHE: Acute Physiology and Chronic Health Evaluation
ATS-IDSA: American Thoracic Society-Infectious Diseases Society of America
BAL: Broncho-alveolar lavage
CI: Confidence interval
CPIS: Clinical pulmonary infection score
ESBL: Extended spectrum beta-lactamase
GLBA: Guidelines-based algorithm
GNR: Gram-negative rods
GPB: Gram-positive bacilli
GPC: Gram-positive cocci
GSBA: Gram stain-based algorithm
ICU: Intensive care unit
IDSA: Infectious Diseases Society of America
MDR: Multidrug-resistant
MRSA: Methicillin-resistant *Staphylococcus aureus*
SHEA: Society for Healthcare and Epidemiology of America
VAP: Ventilator-associated pneumonia
